# P-1983. A Longitudinal Study of COVID-19 Sequelae and Immunity: Effects of SARS-CoV-2 Reinfection

**DOI:** 10.1093/ofid/ofae631.2141

**Published:** 2025-01-29

**Authors:** Paula M Bravver, Bryan Higgins, Michael Sneller

**Affiliations:** National Institute of Allergy and Infectious Diseases, Bethesda, Maryland; National Institute of Allergy and Infectious Diseases, Bethesda, Maryland; National Institute of Allergy and Infectious Diseases, Bethesda, Maryland

## Abstract

**Background:**

Post-acute sequelae of SARS-CoV-2 (PASC) affects approximately 10-30% of COVID-19 patients. Previous research suggests that reinfection with SARS-CoV-2 is associated with worsening PASC regardless of patients’ vaccination status and that reinfection contributes additional risks of pulmonary and other sequelae. The aims of this analysis are to describe rates of SARS-CoV-2 reinfection among patients followed on a longitudinal post-COVID-19 study with different vaccination statuses and compare the effect of reinfection on pulmonary symptoms and function and other PASC symptoms.

**Methods:**

A sub-analysis of a NIAID clinical longitudinal study of COVID-19 was used with a convenience sample of 284 patients infected with SARS-CoV-2. Data was collected at enrollment and at six-month follow-up visits. Reinfection was documented by clinicians during follow-up visits and data on vaccination status was collected at baseline and follow-up visits. Pulmonary function tests including forced vital capacity (FVC), forced expiratory volume (FEV), FEV1/FVC ratio, and diffusing capacity of carbon monoxide (DLCO) were performed annually.

**Results:**

Participants with SARS-CoV-2 reinfection, and those without reinfection, had similar vaccination rates. Among reinfected patients, nearly two thirds of participants reported an improvement in severity or resolution of their PASC symptoms following reinfection. Additionally, no patient without a history of PASC at enrollment developed PASC symptoms after their reinfection. In examining pulmonary function, there was no significant difference in FVC, FEV1, FEV1/FVC, and DLCO changes when comparing patients who were reinfected with SARS-CoV-2 to those who were not.

**Conclusion:**

SARS-CoV-2 reinfection was not observed to be related with vaccine status, was not associated with new or worsening PASC symptoms, and there was no observed decline in pulmonary function when comparing reinfected and not reinfected patients. Continued longitudinal follow-up of this NIAID cohort will help clarify the effect of SARS-CoV-2 reinfection on the natural history of PASC and can inform overall clinical care when treating COVID-recovered patients.

**Disclosures:**

All Authors: No reported disclosures

